# Chronic intermittent hypobaric hypoxia improves markers of iron metabolism in a model of dietary-induced obesity

**DOI:** 10.1186/s12950-020-00265-1

**Published:** 2020-11-07

**Authors:** Fang Cui, Jing Guo, Hao-Fei Hu, Yi Zhang, Min Shi

**Affiliations:** 1 Department of Clinical Laboratory, The Second Hospital of Hebei Medical University, Shijiazhuang, 050000 PR China; 2 Department of Electron Microscope Laboratory Centre, Hebei Medical University, Shijiazhuang, 050017 PR China; 3 Department of Physiology, Hebei Medical University, Shijiazhuang, 050017 PR China

**Keywords:** Chronic intermittent hypobaric hypoxia, Obesity, Iron metabolism, Hepcidin, Erythropoietin

## Abstract

**Background:**

Obesity, a risk factor for many chronic diseases, is a potential independent risk factor for iron deficiency. Evidence has shown that chronic intermittent hypobaric hypoxia (CIHH) has protective or improved effects on cardiovascular, nervous, metabolic and immune systems. We hypothesized that CIHH may ameliorate the abnormal iron metabolism in obesity. This study was aimed to investigate the effect and the underlying mechanisms of CIHH on iron metabolism in high-fat-high-fructose-induced obese rats.

**Methods:**

Six to seven weeks old male Sprague-Dawley rats were fed with different diet for 16 weeks, and according to body weight divided into four groups: control (CON), CIHH (28-day, 6-h daily hypobaric hypoxia treatment simulating an altitude of 5000 m), dietary-induced obesity (DIO; induced by high fat diet and 10% fructose water feeding), and DIO + CIHH groups. The body weight, systolic arterial pressure (SAP), Lee index, fat coefficient, blood lipids, blood routine, iron metabolism parameters, interleukin6 (IL-6) and erythropoietin (Epo) were measured. The morphological changes of the liver, kidney and spleen were examined. Additionally, hepcidin mRNA expression in liver was analyzed.

**Results:**

The DIO rats displayed obesity, increased SAP, lipids metabolism disorders, damaged morphology of liver, kidney and spleen, disturbed iron metabolism, increased IL-6 level and hepcidin mRNA expression, and decreased Epo compared to CON rats. But all the aforementioned abnormalities in DIO rats were improved in DIO + CIHH rats.

**Conclusions:**

CIHH improves iron metabolism disorder in obese rats possibly through the down-regulation of hepcidin by decreasing IL-6 and increasing Epo.

## Background

At present, the incidence of obesity is gradually increasing. Obesity is a risk factor for many chronic diseases, such as hypertension, diabetes, chronic kidney disease and many types of cancer. In recent years, it has been reported that obesity is associated with abnormal iron metabolism [1], and obese people are more prone to iron deficiency (ID) and iron deficiency anemia (IDA) than normal people [2–4]. An estimated 800,000 deaths worldwide are attributed to ID. IDA can lead to fatigue, motor dysfunction, hypothermia, mental retardation, and decreased immunity [1], which is a risk factor for heart failure and increasing mortality. Therefore, the prevention and treatment of obesity and the corresponding ID has very important clinical significance.

Hepcidin regulate the release of cellular iron and body iron homeostasis [5, 6], which is regulated by iron level, inflammation, erythropoietin (Epo) and hypoxia [7, 8] . Recent studies have shown that obesity, a chronic low inflammation state [9–11], upregulates inflammatory cytokine such as interleukin6 (IL-6), then promotes the expression of hepcidin by activating the Janus kinase - signal transducer and activator of transcription-3 (JAK2-STAT3) signal pathway [12, 13] . It has also been confirmed that Epo downregulates hepcidin through the transcription factor CCAAT enhancer binding protein alpha (CEBPA) and homologous DNA binding to the hepcidin promoter binding site [14].

Accumulating evidence has demonstrated the benefits of chronic intermittent hypobaric hypoxia (CIHH) on multiple organs of the body such as heart, brain, liver, and kidney [15–18], including regulating immune system, anti-collagen-induced arthritis [19], antihypertensive activity [15], and improving dyslipidemia and glucose tolerance in type 2 diabetes [16] . Recently, it was reported that CIHH had an anti- aplastic anemia eect through improving the adhesiveness and stress of mesenchymal stem cells [20], and a modulating effect on brain iron homeostasis in rats [18] . Accordingly we proposed the hypothesis that CIHH may ameliorate the abnormal iron metabolism in obesity. In this study, we aimed to investigate the effect and the underlying mechanisms of CIHH on iron metabolism in dietary-induced obesity (DIO) rats.

## Materials and methods

### Animals model establishment and CIHH treatment

Six to seven weeks old male Sprague-Dawley rats (body weight 80–120 g) provided by the Animal Center of Hebei Medical University were randomly divided into chow diet and high-fat-high-fructose diet groups, which were fed with a chow diet and drinking water (containing 22% protein, 4% fat, and 50% carbohydrate; nutrient ratio, specific composition per 1000 g: 99.50 g water, 216.97 g protein, 50.38 g fat, 56.87 g coarse ash, 24.00 g fiber, 13.29 g calcium, 9.17 g phosphorus) and with a high-fat diet and fructose water (containing 24% protein, 12% fat, and 42% carbohydrate; nutrient ratio, specific composition: 8% lard, 2% soy flour and 90% chow diet. 10% (wt/vol) fructose in water) [21], respectively.

After feeding for 16 weeks with different diet, the fat model was established with the following indicator: (Body weight of high-fat-high-fructose diet rat – average body weight of chow diet rat) × 100% / average body weight of chow diet rat ≥20% [22]. Rats were discarded from experiment if their body weight did not reach this threshold. A total of 24 rats were selected and divided into four groups, namely the control (CON), chronic intermittent hypobaric hypoxia treatment (CIHH), dietary induced obesity (DIO), and DIO plus CIHH treatment (DIO + CIHH) group. During the 4 weeks of CIHH treatment (from 17 to 20 week), the rats from the CIHH and DIO + CIHH groups were treated 6 h daily under hypobaric hypoxic conditions simulating an altitude of 5000 m, for 28 days in a hypobaric chamber. The remainder of the time the rats were in a normoxic environment. The rats from the CON and DIO groups were always kept under normoxic conditions. All animals were housed in a temperature-controlled room (22 ± 1 °C) with a 12 h/12 h light/dark cycle, had ad libitum access to food and water, and diet was as usual. During the experiment, body weight and systolic arterial pressure (SAP) were measured at a fixed time every week using a Panlab model LE5001 tail-cuff pressure meter (Harvard Apparatus, Barcelona, Spain).

All the experiments were conducted in compliance with the Guide for the Care and Use of Laboratory Animals (National Research Council 2006), which were reviewed and approved by the Ethics Committee for the Use of Experimental Animals of Hebei Medical University.

### Adipose analysis

At the end of the experiments, rats were fasted overnight and euthanized with a sodium pentobarbital overdose (50 mg/kg, intraperitoneal). Body weight and length were measured to calculate the Lee index (body weight × 1000^1/3^/ length) [23]. Mesenteric, epididymal, and perirenal fats were collected and weighted to derive the fat coefficient (%; (fat weight/body weight) × 100%).

### Blood routine and blood biochemical assay

After the rats were euthanized, 8-ml blood samples were collected from the inferior vena cava of the rats and centrifuged at 3500 rpm for 10 min to obtain serum for assay. The levels of red blood cells (RBCs) and hemoglobin (Hb) were measured with a blood cell analyzer (XS-500i Blood Cell Analyzer; Sysmex, Kobe, Japan). The levels of total cholesterol (TC), triglyceride (TG), high density lipoprotein cholesterol (HDL) and low density lipoprotein cholesterol (LDL) were measured by Colorimetric method. The levels of rat IL-6, Epo and serum ferritin (SF) were measured by ELISA kits (Shanghai Enzyme-linked Biotechnology Co., Ltd., Shanghai, China). The levels of serum iron (SI) and total iron binding capacity (TIBC) were measured by spectrophotometry, and transferrin saturation (TS%) was calculated as SI / TIBC × 100%. Enzymelinked immunosorbent assay (ELISA) was performed to determine the level of IL-6 and Epo according to the instructions of the kit (Shanghai Enzyme-linked Biotechnology Co., Ltd., Shanghai, China), briefly, the serum reacts with the antibody, then absorbance value is detected with a microplate reader at a wavelength of 450 nm followed by the calculation and statistical analysis of concentration from the standard curve.

### Hematoxylin-eosin (HE) staining

Small pieces (1 cm^3^) of liver, kidney and spleen were fixed in 4% paraformaldehyde for 24 h, dehydrated in gradient ethanol step by step, embedded in paraffin, cut into 5-μm thick sections, stained with HE, and then observed under an Olympus BX50 optical microscope (Olympus Optical, Tokyo, Japan). The morphological changes in two sections from each rat, 6 rats in each group were evaluated [19].

### qRT-PCR analysis

Rat liver total RNA was isolated with the RNA extraction kit from Omega Bio-Tek (Norcross, GA, USA), and first-strand cDNA was synthesized using 1 mg total RNA (DNase-treated) using the I script cDNA synthesis kit for reverse transcription purchased from Nanjing Nuoweizan Biotechnology Co., Ltd. (Nanjing, China). qRT-PCR gene expression analysis was performed with the kit for qRT-PCR Master Mix purchased from ABI (Waltham, MA, USA). β-actin was used as an internal control. Primers (Sangong Biotech, Shanghai, China) designed for qRT-PCR gene expression analysis were listed in Table 1. The relative expression of each gene was calculated from 2^-ΔΔCT^. All values were normalized to the levels of β-actin and expressed as relative mRNA level compared to the average level of the CON group.

**Table 1:** Sequence of primers and annealing temperature

Gene	Primer sequence	Annealing temperature
Hepcidin	F 5′- AGATGGCACTAAGCACTCGG − 3′ R 5′- ATCAGCAGCGCACTGTCATC − 3’	56 °C
β-actin	F 5’- GAAATCGTGCGTGACATTAAAGAG − 3′ R 5′- GCGGCAGTGGCCATCTC − 3’	56 °C

### Data analyses

Data are expressed as the mean ± standard deviation (SD), n represents the number of animals in each experiment. Statistical analysis was conducted using one-way analysis of variance (ANOVA) followed by a Student-Newman Keuls’s post hoc test for comparison among multiple groups using the statistical analysis Software Prism 5.0 (Graphpad Software, Inc., La Jolla, CA, USA). A value of *P* < 0.05 was considered statistically significant.

## Results

### Effect of CIHH on body weight and SAP

**Fig. 1:**
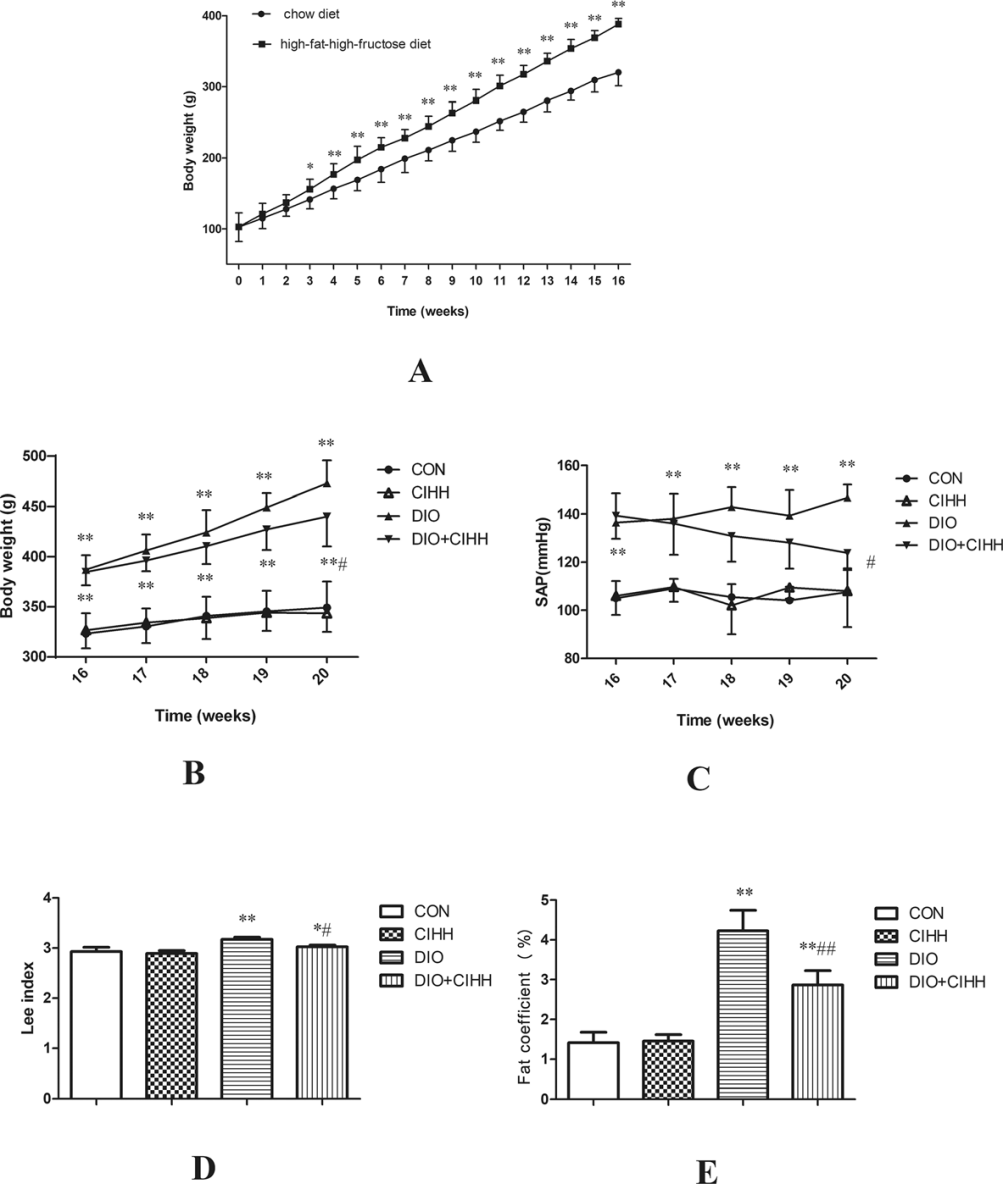
The effect of CIHH on body weight, systolic arterial pressure (SAP) and obesity parameters in rats during CIHH treatment. **a** and **b** Body weight; **c** SAP; **d** Lee index (body weight × 1000^1/3^/ length); **e** Fat coefficient (%; (fat weight/body weight) × 100%). CON: control group, CIHH: CIHH group, DIO: dietary-induced obesity group, DIO + CIHH: DIO + CIHH group. All data are expressed as the mean ± SD; *n* = 5–6 for each group. ^*^P < 0.05 ^**^P < 0.01 vs. CON, ^#^*P* < 0.05 ^##^*P* < 0.01 vs. DIO

During the 16-week period for the development of the obesity model, the body weight of all rats increased steadily (*P* < 0.01; Fig. 1a). After 16 weeks, the body weight of the rats fed with the high-fat-high-fructose diet was heavier than those with the chow diet (320.92 ± 19.69 vs. 388.92 ± 8.24, exceeded 20%, *P* < 0.01; Fig. 1a, b). Even though the diet and water intakes were not different among four groups of rats during the CIHH treatment (data no supply), the body weight of the rats in the DIO + CIHH group was decreased compared with the rats in the DIO group after 4 weeks of CIHH treatment (*P* < 0.05; Fig. 1b).

Before the CIHH treatment, the SAP was higher in DIO and DIO + CIHH rats than that in CON and CIHH rats (*P* < 0.01). After 4 weeks of CIHH treatment, the SAP was decreased in DIO + CIHH rats compared with DIO rats (*P* < 0.05; Fig. 1c).

### Effect of CIHH on obesity parameters

The Lee index and fat coefficient, an index for the visceral fat content and obesity, similar to human’s Body Mass Index, were significantly increased in DIO rats compared with CON rats (3.18 ± 0.04 vs. 2.93 ± 0.09 and 4.23 ± 0.52% vs. 1.44 ± 0.26%, respectively, *P* < 0.01), and were decreased in DIO + CIHH rats compared with DIO rats (3.02 ± 0.03 vs. 3.18 ± 0.04 and 2.87 ± 0.36% vs. 4.23 ± 0.52%, respectively, *P* < 0.01; Fig. 1d, e).

### Effect of CIHH on blood biochemical parameters

#### Blood lipids

**Fig. 2:**
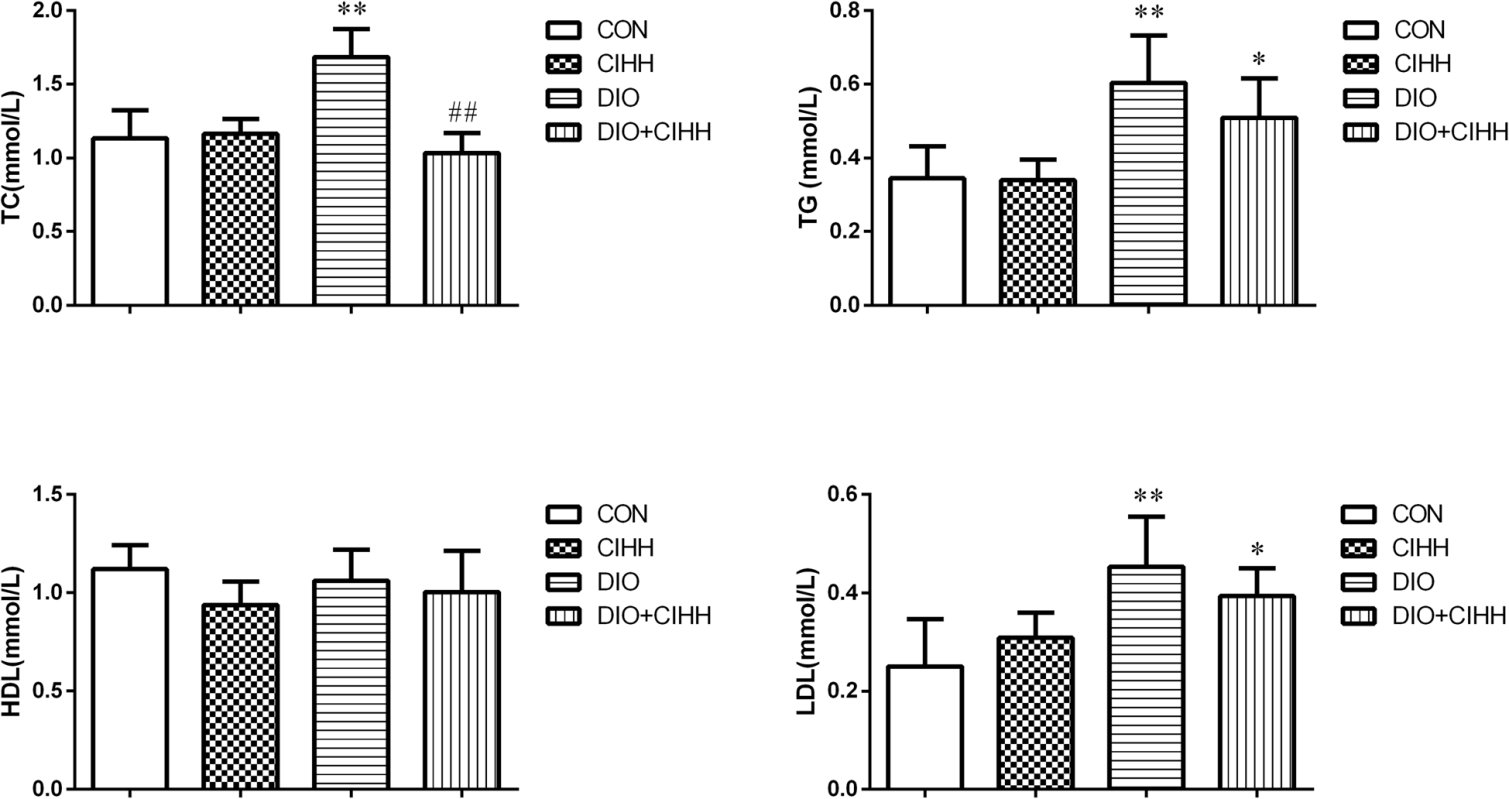
Effect of CIHH on lipid metabolism in rats. TC: Total cholesterol, TG: Triglyceride, HDL: high density lipoprotein cholesterol, LDL: low density lipoprotein cholesterol, CON: control group, CIHH: CIHH group, DIO: dietary-induced obesity group, DIO + CIHH: DIO + CIHH group, All data were expressed as mean ± SD, *n* = 6 for each group, ^*^P < 0.05 ^**^P < 0.01 vs. CON, ^##^*P* < 0.01 vs. DIO

Compared with CON rats, TC, TG and LDL were significantly increased in DIO rats (*P* < 0.01), whereas TC were decreased in DIO + CIHH rats compared with DIO rats (*P* < 0.01), although there were no differences in TG and LDL between DIO + CIHH and DIO rats (*P* > 0.05). There were no differences in HDL among four groups (Fig. 2).

#### Blood routine and iron metabolism parameters

**Fig. 3:**
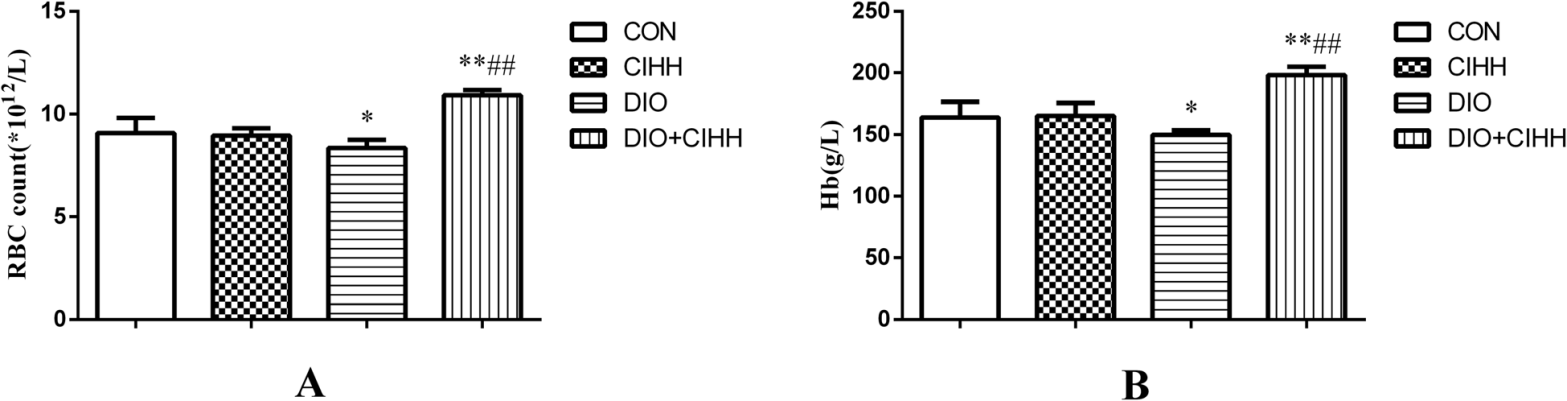
The effect of CIHH on red blood cell and hemoglobin (Hb) in rats. **a** The effect of CIHH on red blood cell (RBC); **b** The effect of CIHH on Hb. CON: control group, CIHH: CIHH group, DIO: dietary-induced obesity group, DIO + CIHH: DIO + CIHH group. All data are expressed as the mean ± SD; *n* = 6 for each group. ^*^P < 0.05 ^**^P < 0.01 vs. CON, ^##^*P* < 0.01 vs. DIO

**Fig. 4:**
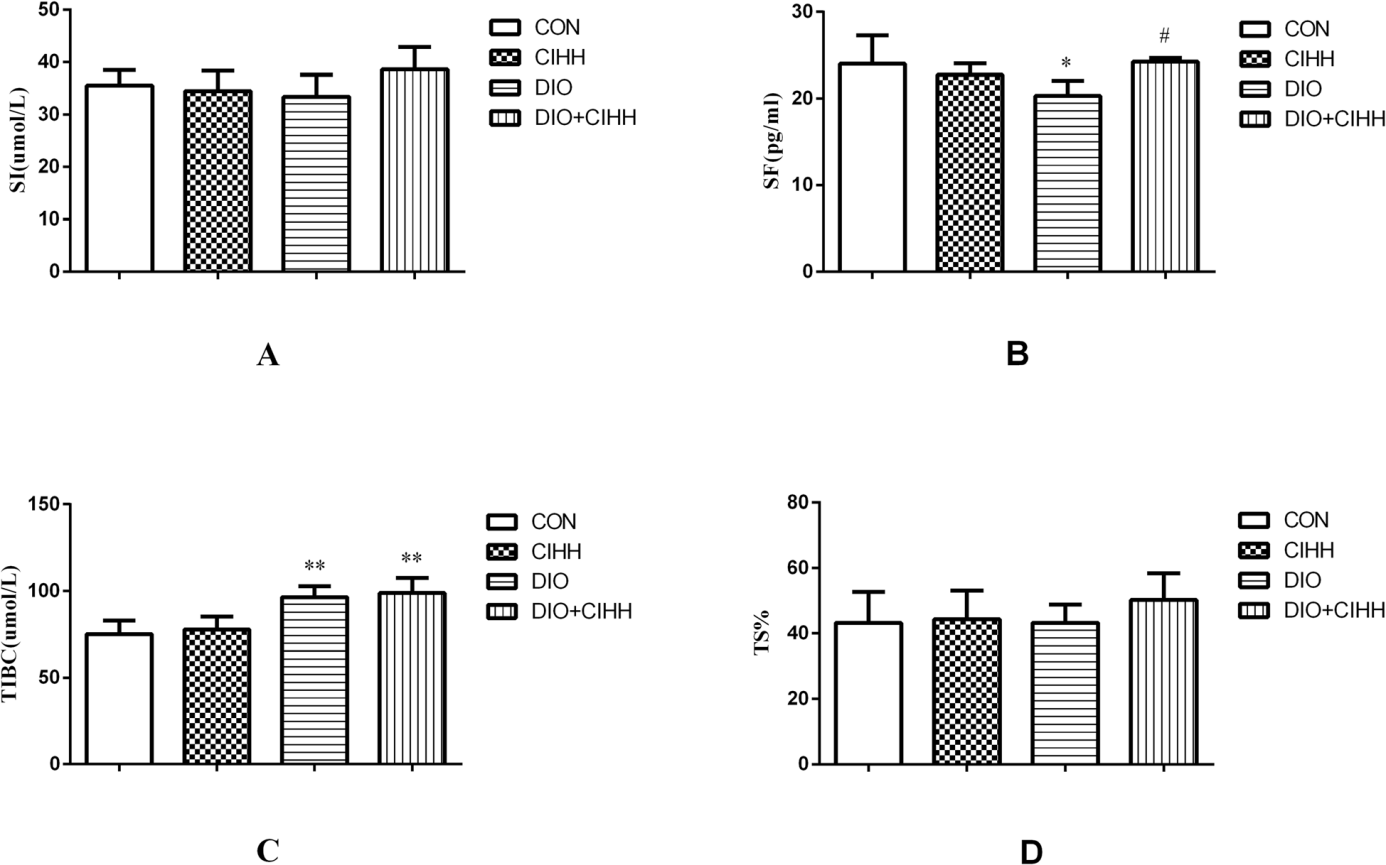
The effect of CIHH on iron metabolism parameters in rats. **a** The effect of CIHH on serum iron (SI); **b** The effect of CIHH on serum ferritin (SF); **c** The effect of CIHH on total iron binding capacity (TIBC); **d** The effect of CIHH on transferrin saturation (TS%). CON: control group, CIHH: CIHH group, DIO: dietary-induced obesity group, DIO + CIHH: DIO + CIHH group. All data are expressed as the mean ± SD; *n* = 6 for each group. ^*^P < 0.05 ^**^P < 0.01 vs. CON, ^#^*P* < 0.05 vs. DIO

RBCs, Hb and SF levels were decreased in DIO rats compared with those in CON rats (*P* < 0.05) and increased in DIO + CIHH rats compared with those in DIO rats (*P* < 0.05; Figs. 3 and 4). The TIBC level was increased in DIO rats compared with that in CON rats (*P* < 0.01), although there was lower in DIO + CIHH group than DIO group, no statistical difference (*P* = 0.592, *P* > 0.05; Fig. 4). There was no difference in the SI level between four groups (*P* = 0.136; Fig. 4).

#### Level of inflammatory factor

**Fig. 5:**
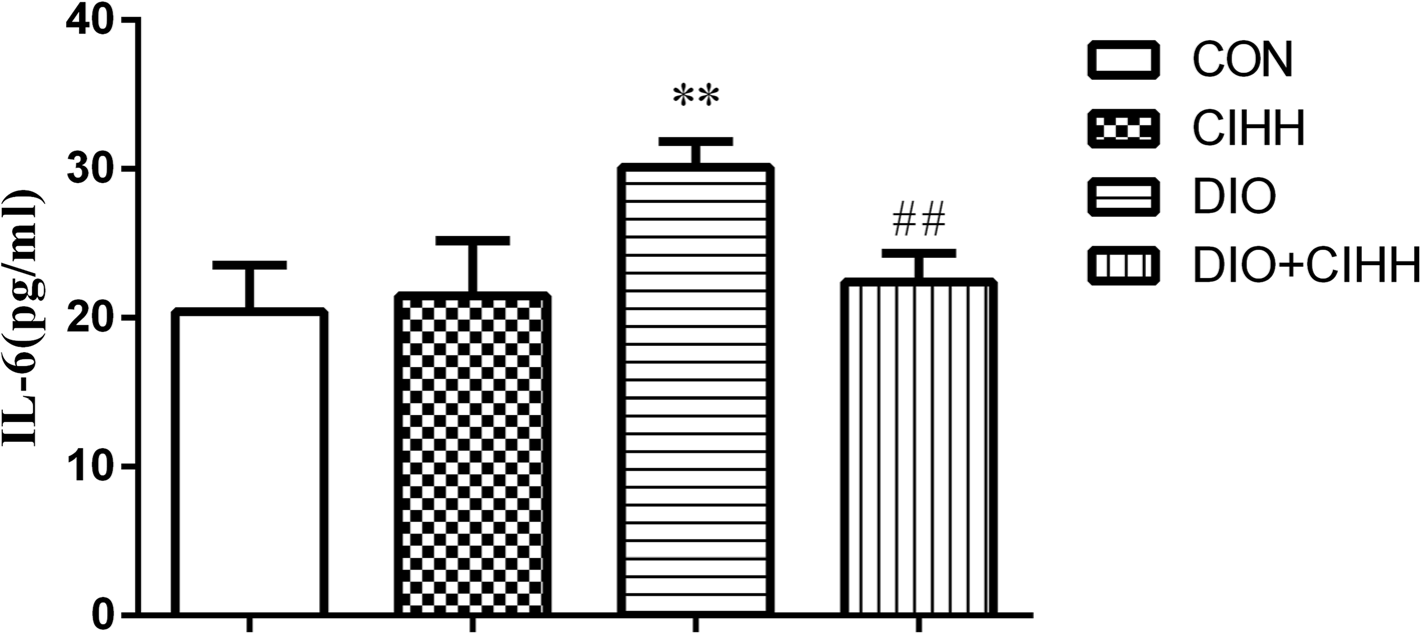
Effect of CIHH on serum inflammatory cytokines IL-6 in rats. CON: control group, CIHH: CIHH group, DIO: dietary-induced obesity group, DIO + CIHH: DIO + CIHH group. All data are expressed as the mean ± SD; *n* = 6 for each group. ^**^P < 0.01 vs. CON, ^##^*P* < 0.01 vs. DIO

Serum IL-6 level, typical inflammatory factor, was increased in DIO rats compared with CON rats (*P* < 0.01) and decreased in DIO + CIHH rats compared with DIO rats (*P* < 0.01; Fig. 5).

### Level of Epo

**Fig. 6:**
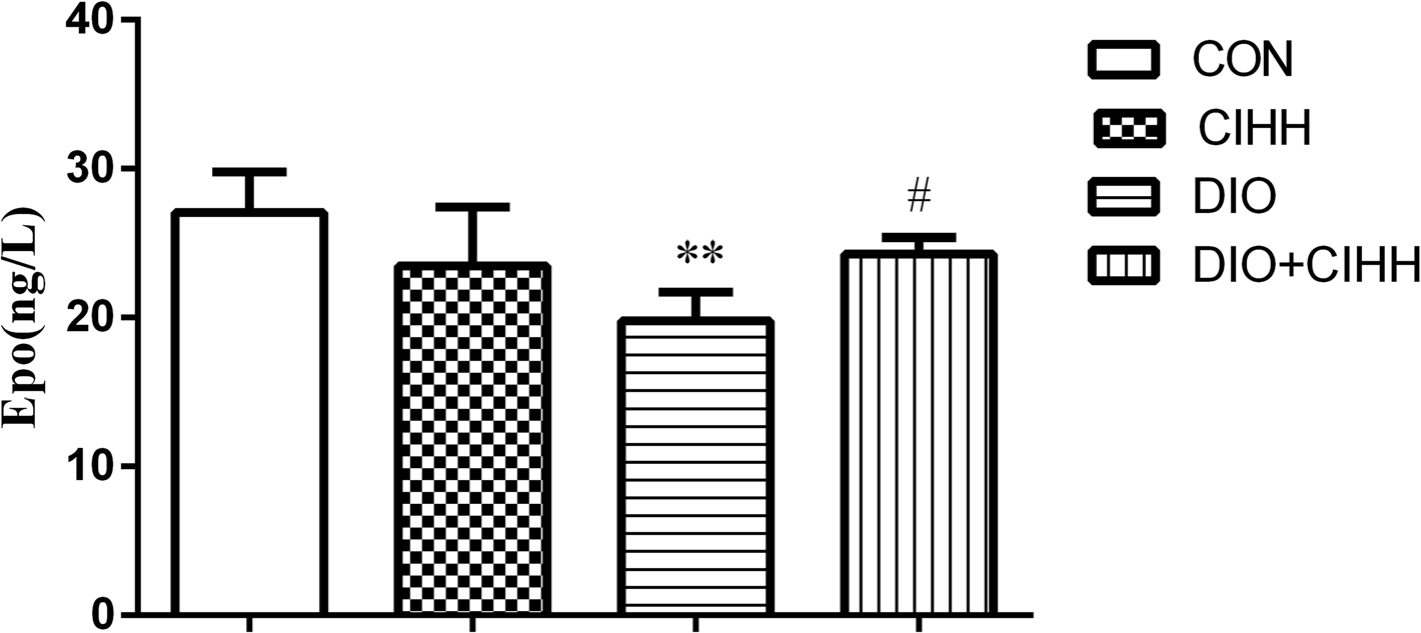
Effect of CIHH on serum erythropoietin in rats. CON: control group, CIHH: CIHH group, DIO: dietary-induced obesity group, DIO + CIHH: DIO + CIHH group. All data are expressed as the mean ± SD; *n* = 6 for each group. ^**^P < 0.01 vs. CON, ^#^*P* < 0.05 vs. DIO

The Epo level was decreased in DIO rats compared with CON rats (*P* < 0.01) and increased in DIO + CIHH rats compared with DIO rats (*P* < 0.05; Fig. 6).

### Effect of CIHH on the histology of liver, kidney and spleen

#### Histological analysis of liver

**Fig. 7:**
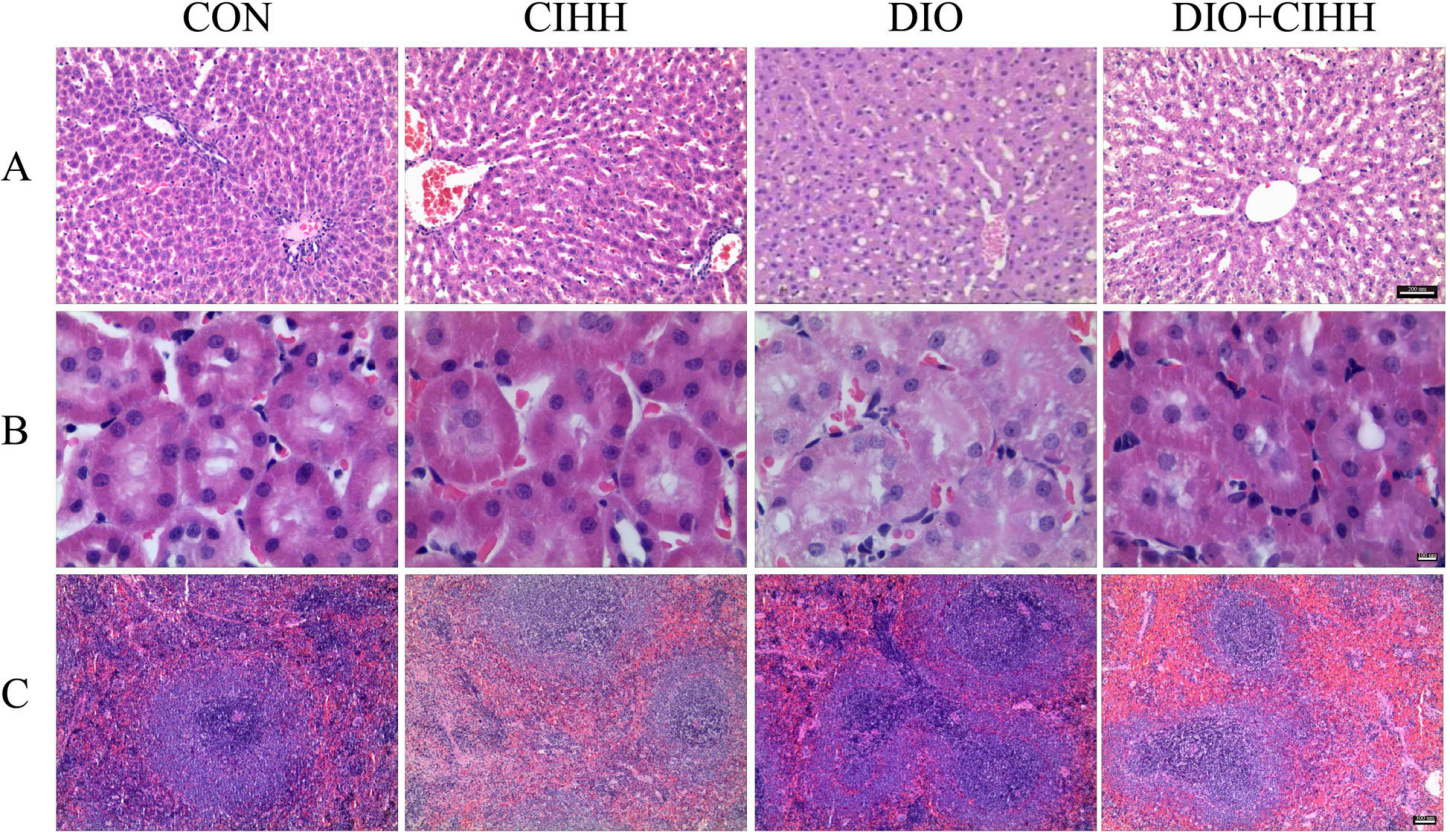
Effect of CIHH on pathological morphology of the liver, kidney and spleen tissues in rats. **a** H & E staining of liver (× 100). There were steatosis, and irregular hepatic cord arrangement in DIO rats; **b** H & E staining of kidney (× 200). There was mild hydropic degeneration of renal tubular epithelial cells in DIO rats; **c** H & E staining of spleen (× 40). There were increased splenic nodules and grown lymphoid tissue in DIO rats. CON: control group, CIHH: CIHH group, DIO: dietary-induced obesity group, DIO + CIHH: DIO + CIHH group. All data are expressed as the mean ± SD; *n* = 6 for each group

Significant hepatic steatosis was found in DIO rats, along with irregular hepatic cord arrangement. The pathological changes were substantially alleviated in DIO + CIHH rats (Fig. 7a).

#### Histological analysis of kidney

Mild hydropic degeneration of renal tubular epithelial cells was found in DIO rats. The pathological changes were considerably alleviated in DIO + CIHH rats (Fig. 7b).

#### Histological analysis of spleen

The splenic nodules were significantly increased in DIO rats, together with the grown lymphoid tissue. The pathological changes were greatly alleviated in DIO + CIHH rats (Fig. 7c).

### Effect of CIHH on the relative mRNA expression of hepcidin

**Fig. 8:**
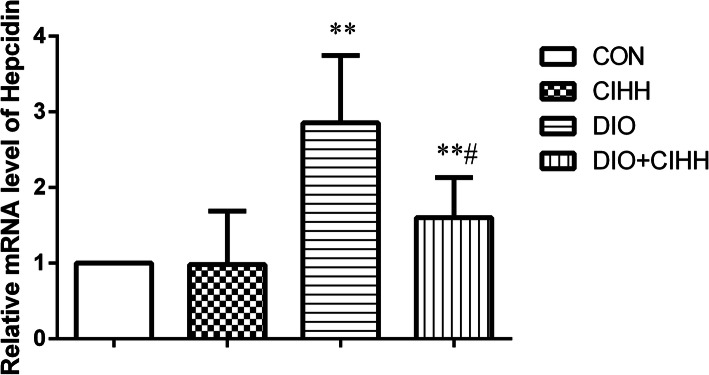
Effect of CIHH on the relative mRNA expression of hepcidin in the liver of rats. CON: control group, CIHH: CIHH group, DIO: dietary-induced obesity group, DIO + CIHH: DIO + CIHH group. All data are expressed as the mean ± SD; *n* = 6 for each group. ^**^P < 0.01 vs. CON, ^#^*P* < 0.05 vs. DIO

The relative mRNA expression of hepcidin was increased in the liver of DIO rats compared with that in CON rats (*P* < 0.01), and was downregulated in the liver of DIO + CIHH rats compared with that in DIO rats (*P* < 0.05; Fig. 8).

## Discussion

In this study, DIO rats exhibited obesity, increased SAP, lipids metabolism disorders, morphological damage of the liver, spleen and kidney, lower levels of RBCs and Hb, and iron metabolic disturbance. Additionally, DIO rats also had increased levels of IL-6 and hepcidin, and decreased Epo level. As we expected, all changes in DIO rats were alleviated by the CIHH, which suggests that CIHH improved iron metabolic disturbance in DIO rats. This improvement might be related to the downregulation hepcidin by decreasing IL-6 and increasing Epo.

Iron is an important trace element that plays a vital role in the transport of oxygen and in erythropoiesis. The imbalance of iron can lead to ID or iron overload [24]. ID initially manifests as iron depletion, then as iron-deficient RBCs, which ultimately leads to IDA [25, 26]. Currently, the commonly used indices to evaluate iron status in the body include RBCs parameters (Hb, RBC count), SF, SI, serum transferrin receptor (sTfR), and TIBC. It has been reported that obesity is often accompanied by abnormalities in iron metabolism, but the results were not completely conclusive. For example, Abo Zeid et al. reported that the levels of SI, TIBC and TS% were lower and SF was higher in obese rats [27]. Khan et al. found a significant decrease in SI and an increase in SF in obese people [28]. Additionally, some researchers reported that both SI and SF were lower in obese rats [29], CIHH had a regulatory effect on brain iron balance, and exerted neuroprotective effects by reducing brain iron content in rats [18]. In this study, we found that there was a significant decrease in Hb, RBC count, SI and SF, but an increase in TIBC in the DIO rats, while an increase in CIHH-treated DIO rats. These results indicate that DIO rats had an abnormal iron metabolism with potential risk of ID, while CIHH had a regulatory effect against abnormal iron metabolism in obese rats.

It is well known that hepcidin is a key regulator of iron homeostasis and in the pathogenesis of anemia [30].^.^Hepcidin regulates plasma iron balance by inhibiting intestinal iron absorption and macrophage iron release [6, 31, 32]. The expression of hepcidin can be induced by inflammation and iron overload, but inhibited by Epo, iron deficiency and hypoxia [7, 8]. Research has confirmed that IL-6 is increased in obesity, a low chronic inflammatory state, and could decrease serum iron level by inducing the expression of hepcidin and downregulating SI via the JAK2-STAT3 signal pathway [12, 13] . It was also reported that moderate normobaric hypoxia could reduce the inflammatory response and the secretion of liver hepcidin, promote intestinal iron absorption, improve the iron homeostasis, and prevent iron deficiency in athletes [33]. The results of this study showed that there was no significant change in liver hepcidin mRNA expression level in the CIHH group, which may be related to the timing of the day and duration of the hypoxia treatment in the animals. In comparison, our results showed the expression of hepcidin mRNA in obese rats was significantly upregulated and the level of serum IL-6 was increased in DIO rats, and the change in hepcidin and IL-6 expression was effectively improved in CIHH-treated DIO rats. Thus, it can be speculated that CIHH improves inflammation and abnormal iron metabolism by inhibiting IL-6-activation and hepcidin in obese rats.

Epo, which is secreted by kidney, regulates the growth of erythroid progenitor cells, and promotes the proliferation of red blood cells and synthesis of Hb. Accordingly, it is considered as another important hepcidin regulator [34]. It has also been confirmed that Epo downregulates hepcidin through the transcription factor CCAAT enhancer binding protein alpha (CEBPA) and homologous DNA binding to the hepcidin promoter binding site [14] . Our previous studies demonstrated that CIHH reduced the hematopoietic dysfunction and antagonized anemia by increasing the serum positive hematopoietic regulatory factor Epo. But it does not clarify the effects and mechanisms of CIHH on hepcidin and iron metabolism in obese rats [20]. In this study, we found that in obese rats the level of Epo was significantly decreased and hepcidin was increased, and CIHH effectively increased Epo and downregulated hepcidin. Thus, it can be speculated that CIHH may inhibit the expression of hepcidin by increasing Epo levels in obese rats. Although it has been reported that injection of synthetic Epo could reduce the level of the inflammatory cytokine IL-6 [35, 36], there are few studies on the relationship between Epo and inflammatory cytokines, and the mechanism is still unclear. Therefore, further studies are needed to establish whether Epo directly inhibits the inflammatory cytokine IL-6 or whether there is an interaction between them, so it warrants further investigation in future studies.

Some research has reported that weight loss, modified diet or physical exercise, decreased the levels of sTFR, IL-6, and hepcidin, while increased the levels of Hb, Epo, and SI [37–39]. In this study, we found that CIHH decreased body weight in spite of no difference in the intakes of diet and water. We are not sure whether weight loss or CIHH treatment or both produce the same results of the iron metabolism, so it warrants further investigation in future studies.

In conclusion, our studies demonstrated for the first time that CIHH improves iron metabolic disturbance in obese rat through downregulation of hepcidin by inhibiting the inflammatory cytokine IL-6 and promoting Epo expression, which implied that CIHH had a regulatory effect on iron balance and exerted protective effects on iron metabolic disturbance of obese rat. Exposure to intermittent hypoxia as a training or treatment method for sports and cardiovascular diseases has been reported decades ago, and has been proved having lots of beneficial action such as losing of body mass and improving metabolic abnormality, decreasing hepatic hepcidin expression and increasing availability of circulating iron that can be used for erythropoiesis [40, 41]. With optimal level and time, CIHH may be promising to become a potential therapy for prevention and treatment of iron metabolic disturbance and ID in anemia patients.

## Data Availability

All results and data are kept in the section for Department of Clinical Laboratory, The Second Hospital of Hebei Medical University, Shijiazhuang, China. These will be made available from the corresponding author on reasonable request.

## Abbreviations

CIHH: Chronic intermittent hypobaric hypoxia
DIO: Dietary-induced obesity
CON: Control group
SAP: Systolic arterial pressure
RBCs: Red blood cells
TC: Total cholesterol
TG: Triglyceride
HDL: High density lipoprotein cholesterol
LDL: Low density lipoprotein cholesterol
SI: Serum iron
SF: Serum ferritin
TS%: Transferrin saturation
TIBC: Total iron binding capacity
IL-6: Interleukin-6
Epo: Erythropoietin
ID: Iron deficiency
IDA: Iron deficiency anemia
Hb: Hemoglobin
ELISA: Enzyme-linked Immune Sorbent Assay
qRT-PCR: Quantitative Real-Time Polymerase Chain Reaction
HE: Hematoxylin and eosin
